# Identifying the location of Cu ions in nanostructured SAPO-5 molecular sieves and its impact on the redox properties†

**DOI:** 10.1039/c8ra10417d

**Published:** 2019-02-22

**Authors:** Jörg Radnik, Thi Thuy Hanh Dang, Suresh Gatla, Vikram Singh Raghuwanshi, Dragomir Tatchev, Armin Hoell

**Affiliations:** Federal Institute for Material Research and Testing (BAM) Unter den Eichen 44-46 12203 Berlin Germany joerg.radnik@bam.de; National Key Laboratory of Petrochemistry and Refinery Technologies, Vietnam Institute of Industrial Chemistry 2 Pham Ngu Lao Hanoi Vietnam; ESRF-The European Synchrotron 71, Avenue des Martyrs 38000 Grenoble France; Humboldt University of Berlin, Institute of Chemistry Brook-Tayler-Str. 2 12489 Berlin Germany; Helmholtz-Zentrum Berlin für Materialien und Energie Hahn-Meitner Platz 1 114109 Berlin Germany hoell@helmholtz-berlin.de; Institute of Physical Chemistry – Bulgarian Academy of Sciences Akad. G. Bonchev Str. Bl. 11 1113 Sofia Bulgaria

## Abstract

Combining X-ray Absorption Fine Spectroscopy (XAFS) with Anomalous Small-Angle X-ray Scattering (ASAXS) determines the location of Cu^2+^ ions in silicoaluminophosphate (SAPO-5) frameworks prepared by hydrothermal crystallization or impregnation. As expected, for the hydrothermally prepared sample, incorporation in the SAPO-5 framework was observed. For the first time preferential location of Cu^2+^ ions at the inner and outer surfaces of the framework is determined. Temperature-Programmed Reduction (TPR) and X-ray Photoelectron Spectroscopy (XPS) investigations demonstrated that such Cu^2+^ is stable in an argon (Ar) atmosphere up to 550 °C and can only be reduced under a hydrogen atmosphere. In contrast, Cu^2+^ deposited by impregnation on the pure SAPO-5 framework can be easily reduced to Cu^+^ in an Ar atmosphere. At lower Cu amounts, mononuclear tetrahedrally coordinated Cu species were formed which are relatively stable in the monovalent form. In contrast, at higher Cu amounts, CuO particles were found which change easily between the mono- and bivalent species.

## Abstract 2

Where is the Cu located? Combining X-ray Absorption Fine Spectroscopy (XAFS) with Anomalous Small-Angle X-ray Scattering (ASAXS) can answer this question for Cu loaded molecular sieves.

## Graphical abstract

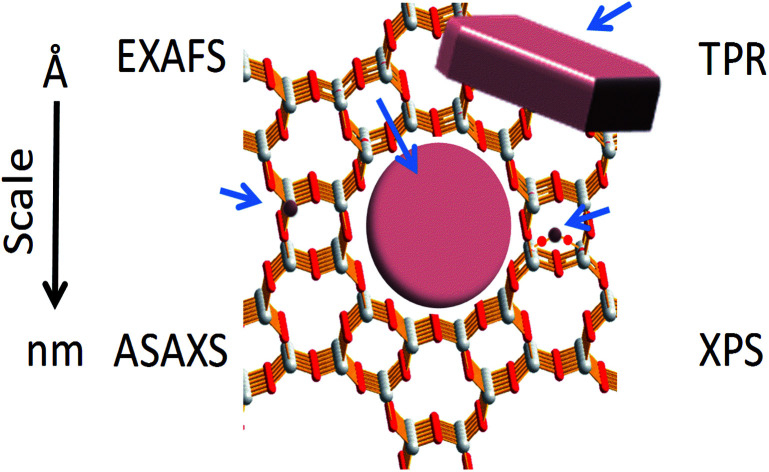

## Introduction

Molecular sieves as supports of transition metal ions (TMIs) offer new opportunities in the preparation of nanostructured materials, because their ordered structure of regular channels or pores in the sub- or low nanometre range allows preparing nanoparticles with more homogeneous size distributions in well-defined sizes. Next to possible applications in the fields like medicine and biology, such modified molecular sieves show promising activity as catalysts in pollution abatement, selective oxidation, and energy conversion.^1–3^ Hereby, not only the kind of metal ion, but also its location and possible clustering influences the properties of the material.^1^ Despite great efforts, the elucidation of structure–properties relationships are still challenging, first of all at the nanometre scale.^4^ Recently, background analysis of X-ray scattering was used for the mesoscale characterization of SBA-15 supported Cu particles and compared with results obtaining from electron tomography.^4^ Another approach used synchrotron powder X-ray Diffraction (XRD) analysed with Rietveld/maximum entropy method to establish structural models of the Cu^2+^ centres in a CHA zeolite.^5^ Herein, we present a novel experimental approach by combining X-ray Absorption Fine Spectroscopy (XAFS) and Anomalous Small-Angle X-ray Scattering (ASAXS) to identify the location of TMIs in such nanostructured molecular sieves.

To show the potential of our approach we have chosen Cu containing SAPO-5 as a model system. Molecular sieves of aluminiphosphate (AlPO) and their isomorphous derivates such as silicoaluminophosphates (SAPO) have been attracted a lot of interest because of structural and compositional diversity which offers unique possibilities to tailor the properties of such molecular sieves.^6,7^ Among of them, the Cu containing SAPO-5 have a potential to be used as catalysts in different reactions, *e.g.* the reduction of NO_*x*_ in presence of hydrocarbons or ammonia (selective catalytic reduction)^8,9^ or the oxidative carboxylation of methanol to dimethylcarbonate.^10^ For this later reaction, investigations at similar systems suggested the formation of CuO_*x*_ agglomerates seems to be beneficial for the catalytic performance due to their higher redox activity compared with species consisting of only a few Cu atoms or incorporated in the framework.^11^ Summarizing, it is out of question, that the local environment of the TMIs has a crucial impact on the catalytic performance.^12^

**Fig. 1:**
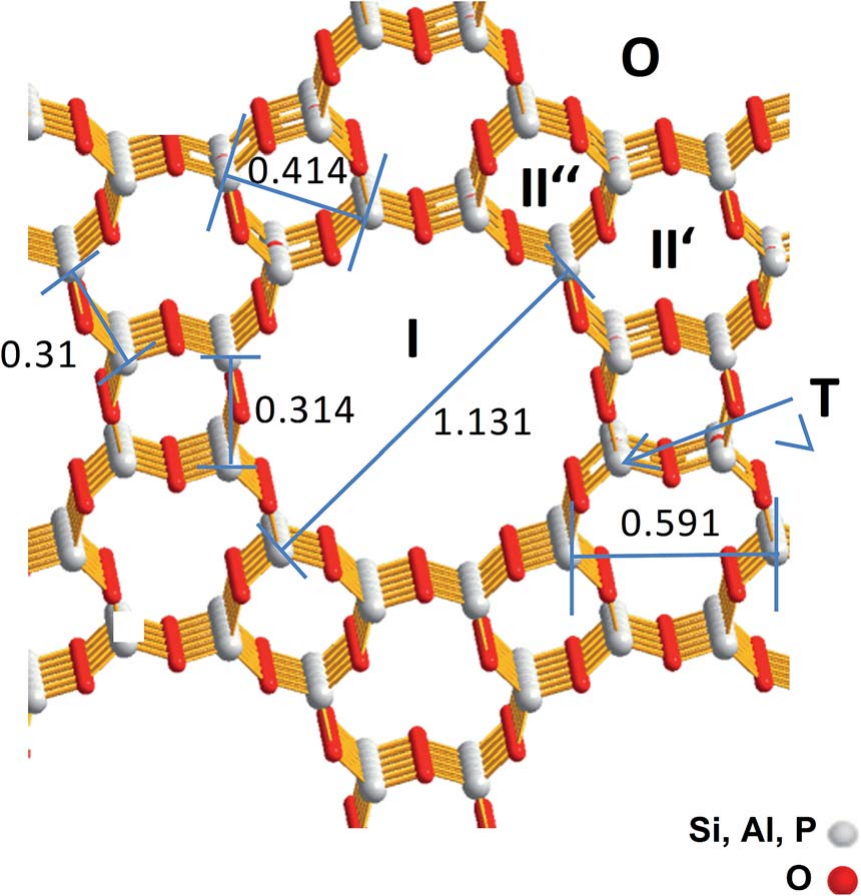
Framework structure of SAPO-5 with the size of the channel like structures and the possible Cu locations (O, I, II′, II′′ and T). T denotes a framework cation position that should be substituted by a Cu ion. Greyish: framework cations (Si^4+^, Al^3+^, P^5+^), red: O^2−^. The distances are represented in nm.

For SAPO-5 with the framework type AFI four different possible Cu locations can be expected which are imaged in Fig. 1.^13^ Cu containing particles could be located on the outermost surface (O) or in the large 12-ring pore with a maximum size of 0.8 nm (I), whereas in the smaller channels (II′ or II′′) only Cu components with very few Cu atoms could be expected. Furthermore, a framework cation (T) can be substituted by one Cu atom. Thereby, electrostatic linking between Cu^2+^ cations in the pores and the lattice and coordinative bonds between Cu^2+^ cations in the pores lattice oxygen were discussed.^9^ Successful incorporation of Cu^2+^ into the lattice was reported for CuAPO-5 after hydrothermal synthesis.^14^

To elucidate the local atomic environment of the TMIs XAFS or Electronic Paramagnetic Resonance (EPR) are the methods of choice.^3,8,14–21^ Both methods allow insight into the valence state and the coordination of the TMIs and their nearest surrounding. Whereas, EPR is only sensitive for ions with unpaired electrons and, herewith, for a limited number of TMIs, XAFS can be applied for many elements in all oxidation states, but requires synchrotron radiation. A common disadvantage of both methods is, that at high TMI loadings the spectra are superposed from ions on different sites, which complicates a straightforward interpretation.^1,22^ Another problem of these methods is the nearest surrounding below 0.5 nm can be investigated very well, but the larger agglomerates between 0.5 nm and 1 nm typical for particles coordinated in channels or cages of the framework cannot be studied. Other structure-sensitive methods like XRD or Transmission Electron Microscopy (TEM) are often not useful for the determination of sites of the TMI. With XRD only the framework or agglomerates with a size of nanometres can be detected, whereas small particles in the channels or cages or the incorporation of TMIs in the framework are usual not visible. One problem of TEM is the high-energy electrons can lead to undesired structural changes of the sensitive molecular sieves. Additionally, with TEM only a few femtograms of materials are measured, but catalysts are used in the order of kilograms or tons, thus TEM investigations are often not statistically and conclusive and need to be checked by a complementary bulk methods.^4^

Small-Angle X-ray Scattering (SAXS) is a powerful method to identify the particles of different sizes and their distribution in any kind of matrices. But conventionally using a monochromatic fixed-energy X-ray source, it is not possible to distinguish between different contributions, *e.g.* the TMI particles and the microporous carrier. To overcome this problem anomalous or resonant behaviour of the atomic scattering amplitude of an element near its absorption edge can be used to separate its scattering contribution from that of the other elements in the sample. This principle of ASAXS was successfully used in the investigation of metal organic networks and supported catalysts.^23–25^ The combination of XAFS and ASAXS allows the structure determination from the atomic range up to several nanometres. Especially, for complex nanostructured systems consisting of several components the element specificity of these methods allows to elucidate the contribution of a specific element to the structure of the whole system, *e.g.* the location of TMIs in microporous materials.

In the following study the structure sensitive methods XAFS and ASAXS were combined to elucidate the location of the Cu ions in the molecular sieve depending on the preparation method. Temperature Programmed Reduction (TPR) and X-ray Photoelectron Spectroscopy (XPS) measurements were performed additionally to reveal the redox properties of the Cu ions in different locations.

## Experimental

### Preparation

The samples were synthesized by hydrothermal crystallization (denoted as SAPO-5 and *x*CuSAPO-5) and by impregnation of pure SAPO-5 with different amounts of Cu containing salt (labelled as *x*Cu/SAPO-5). *x* denotes the wt% of Cu in the final sample determined with inductively coupled plasma optical emission spectroscopy (ICP-OES).

Pseudo-boehmite (Pural SB, purity >99%, Sasol Germany), *ortho*-phosphoric acid (85 wt%, Acros Organics), tetraethyl orthosilicate (TEOS, purity >99%, Sigma-Aldrich) and triethylamine (TEA, purity >99.5%, Sigma-Aldrich) were used as the Al-, P-, and Si-structure directing agent resources, respectively. Certain amount of these components was mixed or dissolved in deionized water and successively added in a beaker after each 60 minutes under continuous vigorous stirring at room temperature to obtain a homogenous gel. The Si : (Al + P) ratio is about 0.1. For the Cu containing sample copper nitrate (Cu(NO_3_)_2_·3H_2_O) (Sigma Aldrich) was added additionally to the former described reaction solution the following hydrothermal crystallization was carried out under autogenous pressure in a microwave oven “Mars5 plus” (CEM, 600 W) in two steps described in detail elsewhere.^26^ After this procedure the solids were filtered, washed and dried at 100 °C for 12 h. Finally, these solids were calcinated at 550 °C for 12 h. The low amount of 2.3 wt% Cu^2+^ allows to reduce a destructive impact of its possible incorporation on the framework.

The Cu-impregnated SAPO-5 samples were prepared by incipient wetness impregnation of calcined SAPO-5 with an aqueous solution of Cu(NO_3_)_2_. The Cu content were adjusted to correspond to that in hydrothermal crystallized 2.3CuSAPO-5 (2.3 wt%) and to a significantly higher amount (9.6 wt%). After drying, the samples were calcinated in air at 450 °C. The notation of the samples is summarized in Table 1.

**Table 1:** The four prepared molecular sieves

Sample	Cu-content/wt%	Preparation method
SAPO-5	0	Hydrothermal
2.3CuSAPO-5	2.3	Hydrothermal
2.6Cu/SAPO-5	2.6	Impregnation
9.6Cu/SAPO-5	9.6	Impregnation

### Characterisation

The following methods were used to analyse the Cu locations.

XAFS experiments at the Cu K-edge (8979 eV) for the 2.3CuSAPO-5, 2.6Cu/SAPO-5 and 9.6Cu/SAPO-5 materials were performed at the BM23 beam line of the ESRF (Grenoble, France). XAFS spectra were recorded in transmission mode for all the samples. As reference spectra, Cu foil, Cu_2_O and CuO were measured. Monochromatic X-ray beam was obtained from the white beam by using Si(111) double crystal monochromator; a harmonic rejection has been performed using Si coated mirrors. Both the incident (*I*_0_) and transmitted (*I*_1_) monochromatic beam intensities were measured by using ionic chambers filled with 1.7 bar N_2_ and 0.3 bar Ar, respectively, and eventually the chambers filled up to 2 bar with He. The photon energy was calibrated with the Cu foil K-edge energy. The samples were pressed into pellets of 13 mm of diameter, whereas for Cu_2_O and CuO references cellulose was used as binder and diluting. The Extended X-ray Absorption Fine Structure (EXAFS) parts of the spectra were collected with a variable sampling step in energy with Δ*k* = 0.05 Å^−1^ up to 15 Å^−1^. For each sample, three consecutive EXAFS spectra have been collected and been averaged for data analysis. The extraction of *χ*(*k*) function has been done using Athena and EXAFS data analysis has been performed with Artemis software.^27^ Fourier transform of EXAFS function *χ*(*k*) into *R* space with *k*^3^ weighted factor and Kaiser–Bessel window function has been performed in 2 to 15 Å^−1^, yielding a function *l χ*(*R*)*l* (Å^−4^). The input files, which contain the information like crystal structure, lattice parameters and space group, given to Artemis were taken either from ATOMS or ICSD (Inorganic Crystal Structure Database).

ASAXS measurements at the Cu-K edge are used to analyses the internal nanoscale structure sizes and to identify the spatial distribution of Cu atoms/ions within the different nanostructure components. ASAXS experiments have been performed on the 7T-MPW-SAXS beamline at the BESSY II synchrotron facility of the HZB. The source is a superconducting 7T-Wiggler.^28,29^ The X-ray beam is focused and monochromatized by a fixed exit system of Si(111) double crystal monochromator having a sagittal bendable second crystal and two flat bendable mirrors.

All four samples were mounted between scotch tapes on a sample changer under vacuum together with a blank scotch tape (background), a glassy carbon scattering standard as a scattering cross section reference and silver-behenate (Ag[CH_3_–(CH_2_)_20_–COO]) as a *q* vector reference. The SAXS instrument is directly attached windowless behind the sample chamber. The small-angle scattering was recorded using an area sensitive Ar gas-filled multiwire proportional counter (200 × 200 mm^2^). These raw data were corrected for dark current, dead time, and sensitivity of the detector as well as by the incoming photon-flux, sample transmission, scattering background, and geometrical effects like the projection of the detector plane on the sphere with radius equal to the sample-detector distance. The relative sensitivity of each detector pixel was determined by collecting the isotropic fluorescence of an appropriate metallic foil (Zn foil at the Cu-K edge) and subtracting the small-angle scattering of this foil measured below the Zn-K absorption edge. The scattering background coming from all components in the beamline was measured before each sample run by moving the sample out of the beam. The sample transmission was measured directly before each scattering measurement using an X-ray sensitive diode (Hamamatsu S2387-1010R). This scattering and sample transmission sequence was repeated at any of the four chosen X-ray energies (8597, 8933, 8957 and 8975 eV) for ASAXS. The whole sample and energy sequence was three times repeated by statistical reasons. The data reduction was done by an instrument specialized data reduction software “SASREDTOOL” to get the final scattering curves. For the data analysis SASfit^30^ was used to get the structural model. It turned out the partial structure could be modelled by a formfactor of homogeneous spheres and a Voigt behaviour of the scattering peaks that are arising from the regular framework structure. Further analysis of SAXS and ASAXS and some necessary theory like the calculation and analysis of the resonant scattering functions are described in the ESI.†

The reducibility of the Cu species was investigated by means of TPR using a gas flow system described in detail elsewhere.^31^ The measurements were performed at samples after calcination, as described above, and after additional treatment in Ar for further 6 h. The calcined and the additionally Ar-treated samples were heated once more *in situ* at 400 °C in an air or at 600 °C in an Ar flow, respectively, for 30 min and then cooled down to 50 °C in a dried nitrogen flow before starting the H_2_-TPR experiment in 5.15 vol% H_2_/Ar flow up to 550 °C with a heating rate of 10 °C min^−1^. Before cooling down to 50 °C in a dried nitrogen flow, the samples were remained for 30 min at this high temperature.

For the XPS experiments the heating procedures were performed under same conditions as described for TPR. After each treatment step (calcination in air at 550 °C and thermal treatment in Ar at 650 °C) the sample were removed from the oven and built in the vacuum system of the XPS equipment with air contact less than 5 min. The XPS measurements were made with an ESCALAB 220iXL (ThermoFisher Scientific) using monochromatic Al Kα radiation (*E* = 1486.6 eV). The binding energies were referred to the C1s peak of adventitious carbon at 284.8 eV. For determining the peak maxima and the areas under the peaks the spectra were fitted with Gaussian–Lorentzian curves after subtracting a Tougaard background. The uncertainty for determining the binding energy is ±0.2 eV with a confidence level of 95%.

## Results and discussion

For a first overview, X-ray Diffraction (XRD) patterns of the pure SAPO-5 and the three Cu containing SAPO-5 samples were obtained. All showed similar patterns typical for the AFI structure. No hints for Cu-containing phases were found for any samples. Only, in case of 2.3CuSAPO-5 an additional very weak reflection appeared which could be correlated to a triclinic AlPO_4_ [PDF-No. 50-54] or with the SiO_2_ polymorph tridymite [PDF-No. 83-1339] (see Fig. S1†). Therefore, the presence of Cu has an insignificant effect on the framework.

### X-ray absorption fine structure

**Fig. 2:**
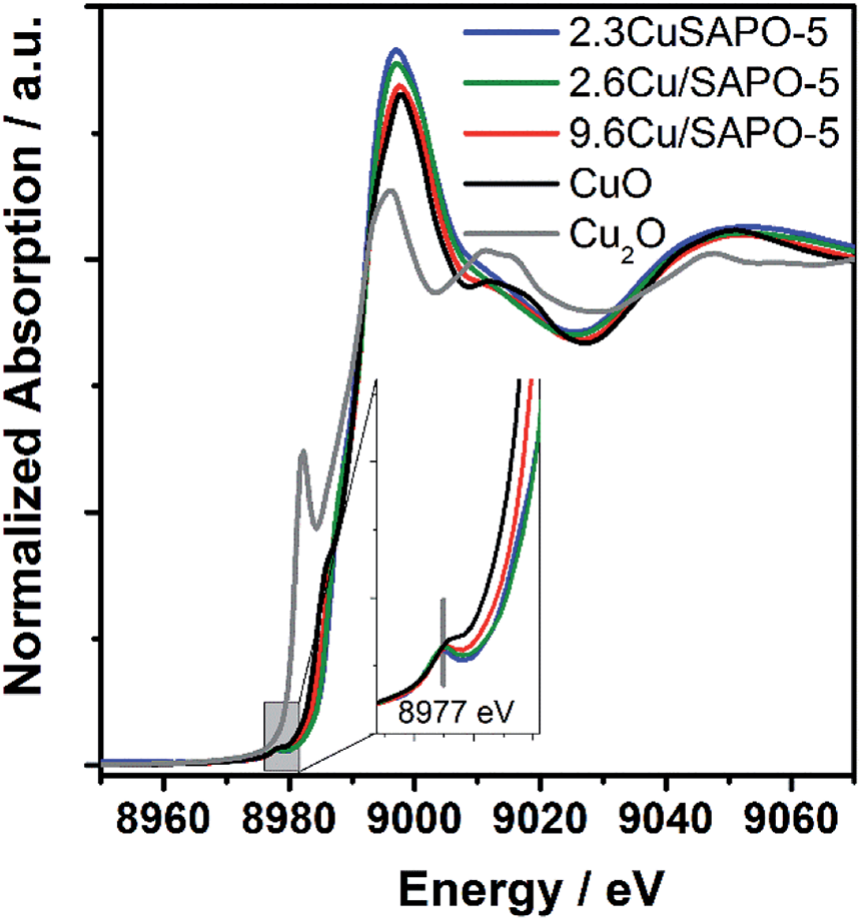
XANES spectra of 2.3CuSAPO-5, 2.6Cu/SAPO-5, 9.6Cu/SAPO-5 and as references CuO and Cu_2_O.

To elucidate the atomic environment of the Cu atoms all three Cu-containing samples were investigated by X-ray absorption methods in the Cu-K absorption edge region. The Near-Edge Structure (XANES) gave first hints for different surroundings of the Cu ions depending on the Cu content and the preparation method. The spectra of all three samples are more similar to CuO than Cu_2_O showing the bivalent character of the Cu ions (Fig. 2). The small pre-edge feature at 8977 eV due to the 1s → 3d transition^32^ characteristic for Cu^2+^ was slightly more pronounced for 2.3CuSAPO-5 and 2.6Cu/SAPO-5 than for 9.6Cu/SAPO-5 or CuO, which could be explained by a more distorted octahedral coordination of the Cu ions at the low loadings than at 9.6Cu/SAPO-5 or at CuO.^33^ For all Cu containing SAPO-5 samples, a shift to higher absorption edge energies were observed compared to CuO: for the two samples with a lower Cu content the absorption edge shifted 2.5 eV higher, and for the 9.6Cu/SAPO-5 sample 2 eV higher. XANES observations can predict the coordination of the Cu ions in the lower loaded samples differ more significantly than in the 9.6Cu/SAPO-5 sample from CuO. However, only a detailed EXAFS analysis can cast a light on the differences in the atomic structure.

**Fig. 3:**
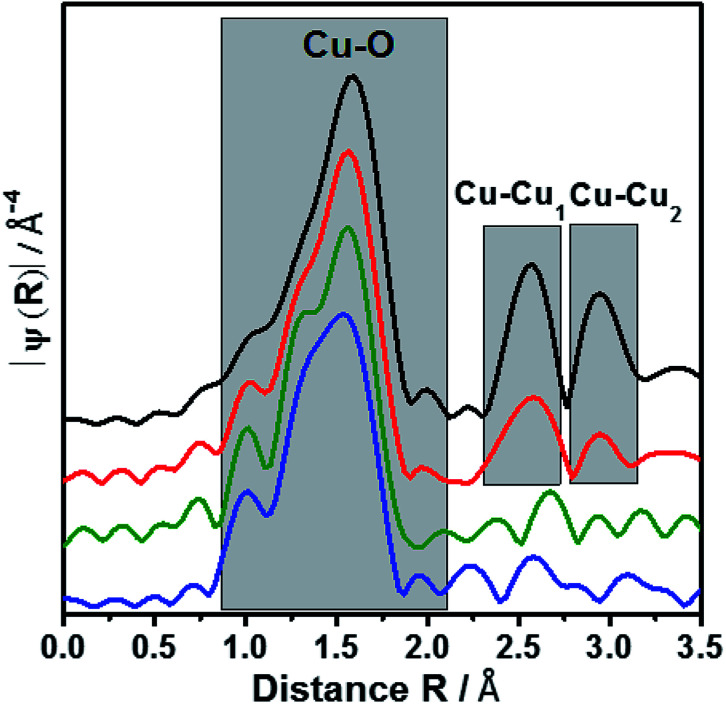
Fourier transform of EXAFS oscillations of 2.3CuSAPO-5, 2.6Cu/SAPO-5, 9.6Cu/SAPO-5 and CuO reference. The colour scheme follows Fig. 2.

EXAFS oscillation can be clearly seen up to 15 Å^−1^ (Fig. S2†). Especially, at larger *k* values, where higher coordination distance reflects, the variations are more apparent. Fig. 3 showed the Fourier transform of *k*^3^-weighted EXAFS spectra of different samples 9.6Cu/SAPO-5, 2.6Cu/SAPO-5 and 2.3CuSAPO-5 and the CuO reference compound. The shaded region in this figure belongs to the Cu–O distances in the first coordination shell. Fitting analysis for the CuO reference reflect clearly the four oxygen atoms of the first coordination shell in the x-y plane at 1.93 Å and 1.96 Å, whereas two axial oxygen atoms around 2.4 Å are end up with a large Debye–Weller factor and a high error range (Table S1†). This result fits with the tenorite structure for CuO (ISCD Collection Code 67 850). This later contribution is missing for all the Cu-containing SAPO-5 samples. For the first coordination shell no changes in the coordination number were observed, but for the nearest-neighboured two oxygen-atoms a contraction of the distance was found, interestingly not for the two other oxygen-atoms in the plane (Fig. 3). This contradiction matches with the slight changes in the coordination geometry reflected by the variation of the pre-edges in the XANES. In former studies, lattice contraction was reported for CuO nanoparticles of a few picometers.^34^

As expected, only Cu–O bonds could be observed for the first coordination shell, but only the knowledge about the second coordination shell allows to distinguish between CuO nanoparticle with Cu–O–Cu bonds, mononuclear Cu without such bonds or Cu in the framework with bonding between Cu, O and the framework cations (Si, Al or P).

The 9.6Cu/SAPO-5 sample shows two distinct peaks in the region between 2.25 and 3.25 Å (Fig. 3) which are found for CuO too. This observation showed the presence of CuO particles in this sample. On contrary, samples with lower Cu content contain no such distinct peaks which reveal the absence of CuO particles. In case of impregnated sample (2.6Cu/SAPO-5), the oscillations of the amplitudes are small and do not correspond with any second shell correlation, *e.g.* Cu–Cu or Cu–Si. These observations could be explained with isolated mononuclear tetra coordinated Cu(ii) species. On the other hand, at the sample 2.3CuSAPO-5 synthesized hydrothermally, the intensity and the shapes of the peaks at higher *R* are clearly distinct from the rest of the samples. Contributions from Cu–Si with the distance of 2.9 and 3.13 Å appeared at the corresponding region between 2.3 to 2.9 Å (Fig. 3) indicating that in the second shell framework cations were present. It must be noted that it is not possible to differentiate between Si, Al or P in this evaluation due to their similar atomic number. This observation could be explained with an incorporation of Cu into the framework. For the AFI framework distances between the cations of 3.13 Å and 2.9 Å are described.^13^ This result points to the incorporation of Cu into the SAPO-5 framework while substituting a framework cation. Summarizing, depending on the Cu amount and the preparation method, different Cu components could be created: CuO nanoparticles are only available at high Cu amount, low Cu content leads to species with isolated Cu atoms: after impregnation isolated mononuclear Cu^2+^ species coordinated by four oxygen atoms with no hints for the second shell in the EXAFS oscillations, whereas the hydrothermal synthesis leads to Cu^2+^ incorporated into the framework.

### Small-angle X-ray scattering

**Fig. 4:**
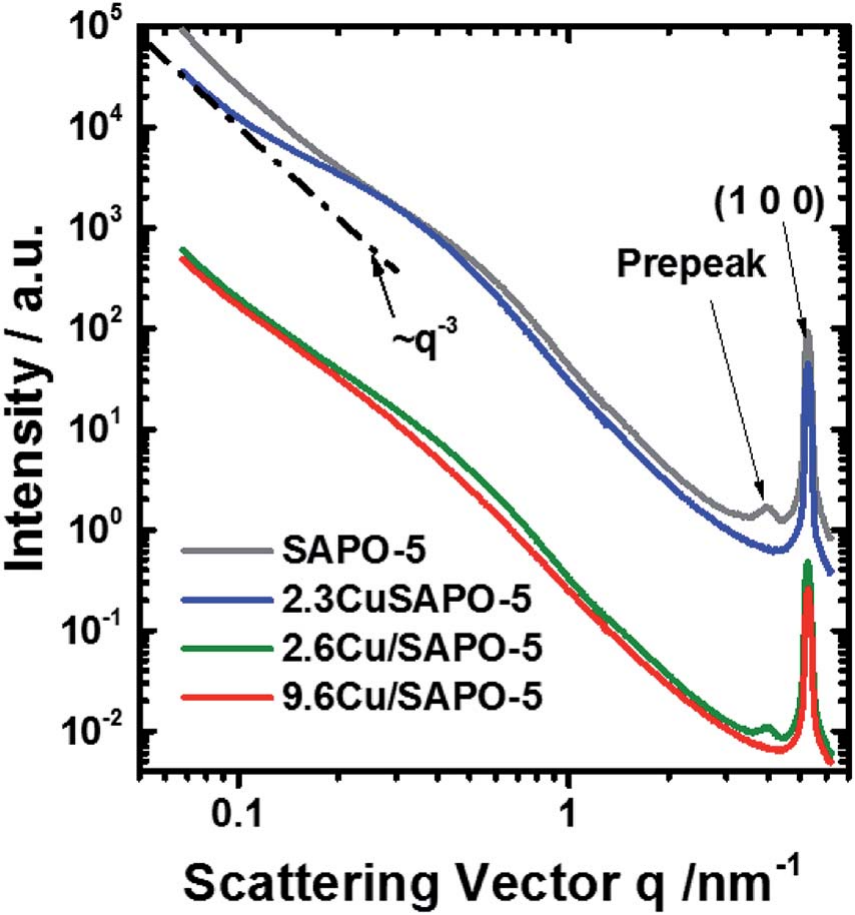
SAXS curves measured at 8579 eV. Some curves are shifted for clarification. The line ∼ *q*^−3^ is shown for illustration.

SAXS and element sensitive ASAXS measurements were performed to evaluate the nanostructural distribution of Cu atoms/ions into the molecular sieve matrix. The scattering curves shown in Fig. 4 of the four samples are very similar and contain up to four elements. A Porod-like power function contribution proportional to the scattering vector, *q*, on power −*n* tends to prevail at lowest scattering angles. A hump adds to that contribution in the *q*-range 0.15 < *q* < 2 nm^−1^. Incoherent scattering background with one or two peaks appear at *q* > 2 nm^−1^. The stronger peak position corresponds to the (100) reflection of the SAPO-5 framework at *q* = 5.275 nm^−1^.§§XRD pattern was simulated with “Database of Zeolite Structures”: http://asia.iza-structure.org/SC/pow_pat.php?STC=AFI&ID=AFI_0”; downloaded at 2018-09-21. All the four mentioned features including the pre-peak are present already in the pure SAPO-5 sample. This means addition of 2.6 wt% Cu does not lead to significant change and the scattering of the Cu containing samples is still dominated by the scattering of the intrinsic SAPO-5 structure. For a powder sample the origin of the scattering are the grain boundaries, internal surfaces and the network of voids between the particles in the sample. Thus, a various cluster and fractal structures should be the relevant model. The analysis of the scattering curves was based on these four contributions and an additional set of monodisperse spherical particles, that would fit the channels along the [100] axis of the SAPO-5 framework. The hump in the range 0.15 < *q* < 2 nm^−1^ was approximated with mass fractal with Gaussian cut-off^35^ and log-normally distributed aggregate size. Software SASfit was used to fit the respective SAXS curves.^30^ Details are given in the ESI.†

The scattering curves fit well to the selected model within the entire *q*-range for all measured energies. A combination of two Voigt functions successfully fit the shape of the (100) peak. The contribution of small monodisperse particles, that would reside in the channels along the [100] axis, may be significant only for the sample with 2.3 wt% Cu. In all other cases, their introduction is irrelevant since the fit is already good and no more information can be further extracted. However, the scattering contribution of the monodisperse spheres appears to be very small and present in the most poorly fitted area of the scattering curves (*q* > 2 nm^−1^). Thus, even for the sample with 2.3 wt% Cu, we should treat the results as a possibility to have small particles in the framework channels along the [100] axis, but not as a proof for their existence. Thus, no attempt to determine their possible anomalous effect was made.

**Fig. 5:**
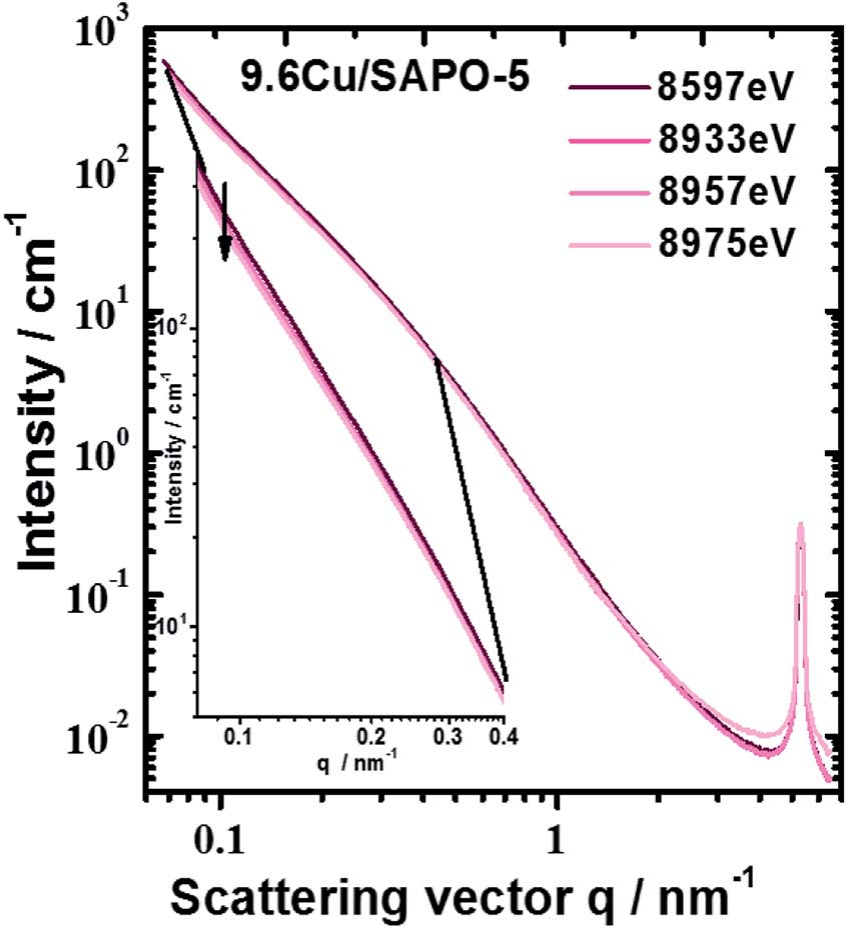
Scattering curves of sample with 9.6 wt% Cu measured at different energies. Largest anomalous effect was observed at smallest scattering angles (see inset).

An anomalous scattering effect is observed only for the sample with 9.6 wt% Cu, as shown in Fig. 5. That is expected since the Cu content is actually very low 0.75, 0.85 and 3.43 at% for the samples with 2.3 2.6 and 9.6 wt% Cu correspondingly. For the 9.6 wt% sample the anomalous effect is however strongest at the smallest angles, which means that the Cu containing entities have dimensions of the order of over 80 nm. The fitting procedure was able to distinguish between the power law and the hump modelled with mass fractal scattering of aggregates. The anomalous effect (Fig. 5) turned out to be due to the power law contribution. Since Porod constant is proportional to the scattering contrast, the anomalous effect can be quantitatively assessed. Thus, a clear anomalous effect is seen only for the power law contribution of the sample with highest Cu content, but it is still smaller than the expected for Cu, Cu_2_O or CuO. An effective Cu content can be determined from the anomalous effect of the sample with 9.6 wt% Cu. Fitting the energy dependence of scattering contrast with artificial molecule consisting of 1 − *x* parts of the basic composition with 0% Cu and *x* parts of Cu one obtains that the large scattering entities should contain about 27 at% Cu. This number is much higher than the added Cu −3.43 at% that means that Cu is not homogeneously distributed within the large entities. Qualitative explanation can be given by assuming a core–shell particle (see the ESI†). If the shell contains much more Cu, *e.g.* consist of CuO, the measured energy dependence will be weaker since the Porod constant combines the scattering contrasts of the core and the shell. Therefore, the Cu content in the shell could be higher than 27%, but that of the core – much lower. Keeping in mind the result from the EXAFS, the shell is most probably CuO, but the core contains no Cu or negligible amount of Cu.

The fractal dimension of the aggregates seems to be independent of the Cu addition, but different for the two methods of preparation 2.78 and 2.88 on average for the hydrothermal and impregnation methods correspondingly. The mean radius of the aggregates, assuming spherical shape, and its dispersion grow with the addition of Cu for both types of preparation. However, no anomalous effect can associate with these aggregates and therefore they do not contain detectable quantity of Cu.

The Porod exponent, between 3 and 4 for measurement with point collimation system is most often associated with rough surface of the scattering entities. That applies to the hydrothermally prepared samples. In this case, if surface fractals are assumed the fractal dimension would be 6 − *n*, the corresponding values are shown in Table S2 in the ESI.† For the impregnated samples, the Porod exponent below 3 would suggest again mass fractal with fractal dimension equal to the Porod exponent. The fractal dimensions for both preparation approaches are however very close to the boundary between surface and mass fractals.

To an accuracy of the experiment, both peaks are not connected with the presence of Cu and show no X-ray energy dependence. The disappearance of the pre-peak in the 2.3 wt% Cu sample and his presence in the 2.6 wt% Cu sample leads to conclusion that the two methods of preparation lead to different interaction of the Cu with the SAPO-5 framework. The pre-peak has same position for the two samples. Contrary, the (100) peak of the 9.6 wt% sample tends to shift to higher *q*-values, regardless of the method of determination of its position. Thus, distortion of the crystal structure under incorporation of Cu is present and probably different for the two preparation methods.

Overall, the lack of ASAXS for two of the Cu-containing samples as well as finding ASAXS at large sizes in the third suggests that Cu is mostly homogeneously distributed in the SAPO-5 framework. The small amount of Cu, ASAXS is not observed as could be expected if Cu was clustered into pure Cu or CuO nano-dimensional phase. Exception is the 9.6 wt% Cu sample where the anomalous effect suggests inhomogeneous distribution of Cu associated with large scattering entities over 80 nm. Given the small quantity of Cu, these Cu containing phases, most probably CuO, decorate the surface of the large scattering entities. No proof can be given for Cu-containing particles in the [001] channels of the crystal structure of SAPO-5.

### Redox properties

One major aim of this study is to investigate the influence of the different Cu locations on the redox properties of the Cu ions. For this purpose, the three samples with the different Cu locations were treated with different reduction procedures in inert gas and in a H_2_ atmosphere. The investigations in inert gas are of great interest, because it is known that such thermal treatment leads to the desired Cu^+^ ions which are beneficial for the catalytic properties of the materials.^36^ The investigations in this field were performed with Temperature Programmed Reduction (TPR) and X-ray Photoelectron Spectroscopy (XPS).

#### Temperature programmed reduction

To obtain first insights into the reduction behavior of different samples, TPR investigations in a H_2_ atmosphere were performed for the calcined samples (as investigated in the former sections) and after additional treatment in the inert gas Ar at 600 °C for further 6 h. For the treatment in Ar, the reduction of bivalent Cu to monovalent Cu was proposed, whereas treatment in H_2_ should lead to zero-valent Cu.^36–38^

**Fig. 6:**
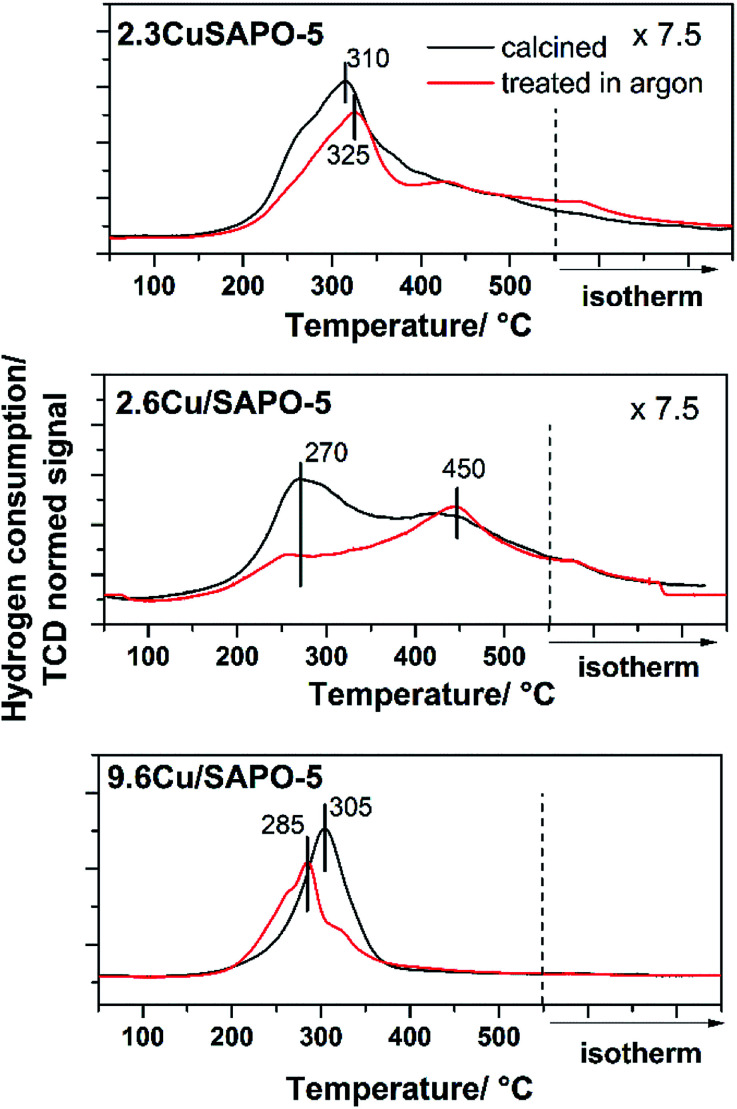
H_2_-TPR profiles of 2.3CuSAPO-5, 2.6Cu/SAPO-5 and 9.6Cu/SAPO-5 after calcination (black) and after calcination plus sequent Ar treatment (red).

The TPR profile of the calcined and Ar treated samples are presented in Fig. 6. Obviously, the different preparation and Cu loading, and hereby, the location, influenced the reduction of the Cu^2+^ ions. For the hydrothermally synthesized sample 2.3CuSAPO-5 a profile with a maximum at 325 °C was observed. After treatment in Ar at 600 °C, the temperature profile changed slightly, and the total hydrogen consumption over the whole temperature range was similar. This can be explained with some slight changes of the Cu^2+^ ions, *e.g.* dehydration, but a reduction of the Cu during the argon treatment can be excluded.

The situation differed for both impregnated samples. For the material with low Cu-loading (2.3 wt%), the reduction occurred in a broad temperature range between 150 °C and 550 °C with two maxima at 250–300 °C and 450 °C. The thermal treatment in Ar clearly diminished the intensity of the peak at 250–300 °C. For CuO it was observed, that Cu^2+^ was preferentially reduced at lower temperatures below 300 °C, whereas monovalent Cu^+^ was reduced at higher temperatures.^34^ The peak profile of the 2.6Cu/SAPO-5 sample suggests a sequential reduction of Cu^2+^*via* Cu^+^ to Cu^0^. The thermal treatment in inert gas led to the reduction of Cu^2+^ to Cu^+^ shown by the decrease in the H_2_ consumption at temperatures below 400 °C. For these both profiles of the low-loaded samples a significant tailing was observed hinting to strong interactions between the Cu ions and the SAPO-5 framework.

For 9.6Cu/SAPO-5 the TPR peak profiles were different. Before and after the treatment, the maxima were around 300 °C, but the hydrogen consumption differed. For the non-treated sample, more hydrogen was needed for the reduction than after the treatment in Ar. Obviously, at least a part of the Cu^2+^ was reduced during the treatment. From these results, it is unclear if a part of the Cu^2+^ was reduced to metallic Cu, or the major part of the Cu^2+^ was reduced to Cu^+^ during this step. For the catalytic activity of the material the latter is desired.^36^ To answer this question and obtain further insights into the redox properties XPS measurements were performed.

#### X-ray photoelectron spectroscopy

**Fig. 7:**
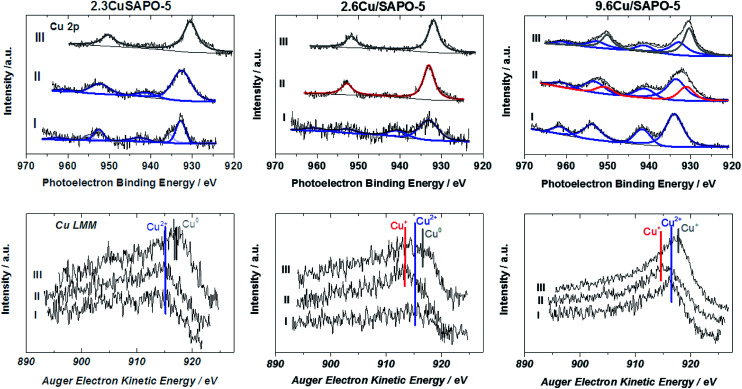
Cu 2p X-ray photoelectron spectra (first row) and the concerning Cu LMM Auger spectra (second row) of the samples before treatment (I), after treatment in Ar (II) and after reduction in H_2_ (III). Blue lines indicate Cu^2+^, red lines Cu^+^ and grey lines Cu^0^.

The valence states of the Cu ions were measured immediately after different thermal treatment procedures with XPS to get a realistic understanding of the reduction process. The spectra achieved in this way were compared with the calcined samples (Fig. 7). It is well known that it is not possible only with Cu 2p spectra to distinguish between mono- and zerovalent Cu due to the similar binding energy for both states. Additionally, the environment of the Cu ions influences the binding energy of the Cu 2p electrons.^39^ For a more reliable determination of the valence states of Cu, in addition, Cu LMM Auger spectra were monitored. Another problem of these samples is the re-oxidation of reduced Cu in air (see ESI†).

The Cu 2p spectrum of the calcined 2.3CuSAPO-5 shows two Cu 2p_3/2_ peaks at 932.9 and 935.4 eV with a weak satellite at 942.6 eV. Neither the Cu LMM Auger spectra nor the Cu XANES results gave any hints for the existence of Cu^+^ or metallic Cu for the samples before the reduction. These peaks were reported for other Cu loaded molecular sieves and attributed to the co-existence of CuO and Cu^2+^ ions.^40^ The peak at 935.4 eV were correlated to hydrated Cu(H_2_O)_6_^2+^ ions and disappeared after dehydration. The Cu LMM spectra confirmed the existence of bivalent Cu. The thermal treatment in Ar did not lead to a significant shift of the LMM peak showing that no reduction occurred during this treatment. No significant shift was observed for the Cu 2p peaks, only a broadening. These observations excluded the reduction of the Cu^2+^ ions during this treatment, which confirmed with the TPR results for this sample. The subsequent H_2_ treatment led to the reduction of bivalent Cu to zerovalent Cu indicated by one Cu 2p_3/2_ peak at 930.5 eV and a shift of the Cu LMM Auger peak to a higher kinetic energy.

The situation differed for the impregnated sample 2.6Cu/SAPO-5 having a similar Cu content as the hydrothermal synthesized one. At the calcined sample, one Cu 2p3/2 peak at 933.1 eV with a strong satellite at 941.5 eV occurred. After the treatment in Ar the satellite disappeared. Simultaneously, the Cu LMM Auger peak shifted to lower kinetic energies. This could be explained by the reduction of Cu^2+^ to Cu^+^, which was observed with the TPR investigations, as well. Further treatment with H_2_ led to the reduction to metallic Cu. For the higher-loaded 9.6Cu/SAPO-5 material the reduction behaviour was comparable. Treatment in Ar led to a partial reduction of Cu^2+^ to Cu^+^ and the following reduction in H_2_ to metallic Cu. In each sample differently intense satellites typical for CuO were observed. From the XPS observations it is still unclear, if only a part of Cu^2+^ is reduced or the reduced Cu was re-oxidized during the short air contact. The TPR results indicating an oxidation state change of two for the calcined sample supported the latter hypothesis.

Obviously, the preparation method, and herewith the location of the Cu ions as identified by the combination of ASAXS and XAFS, influences the redox properties of these ions. Only at the impregnated samples with isolated Cu ions or CuO particles a reduction from bivalent Cu to monovalent Cu was observed, whereas Cu^2+^ ions incorporated in the SAPO-5 framework cannot be reduced to Cu^+^, only to metallic Cu. The high-loaded sample with the CuO nanoparticles can change between the valence states +2, +1 and 0 most simply.

## Conclusions

Combining the element-specific methods EXAFS and ASAXS offers unique possibilities to elucidate the Cu^2+^ location in a SAPO-5 molecular sieve. At low contents, randomly distributed Cu ions were found. EXAFS revealed that the Cu ions were incorporated in the framework with the hydrothermal method and located in the pores of the material after the impregnation. Impregnation with higher amounts of Cu led to the formation of a CuO shell at the outer surfaces, preferentially, which was shown by the ASAXS investigations.

As expected, the position influences the redox properties of the Cu^2+^ ions which was investigated with TPR and XPS measurements after reducing the Cu^2+^ ions in Ar and H_2_. Whereas, the incorporated bivalent Cu is stable and can be only reduced under harsh conditions to metallic Cu, the valence states of the ions in the Cu particles can be switched between the bi- and monovalent oxidation state. For preparing relatively stable monovalent Cu the randomly distributed single Cu ions are most suitable, whereas Cu ions in the shell can easily change between the both oxidation states. Thus, depending on the preparation method and the Cu content materials with the desired redox properties can be tailored.

## Conflicts of interest

There are no conflicts to declare.

## Supplementary Material

RA-009-C8RA10417D-s001
