# Supplementary material for: Identifying the location of Cu ions in nanostructured SAPO-5 molecular sieves and its impact on the redox properties
Source: RSC Adv. 2019 Feb 22;9(12):6429–37. doi: 10.1039/c8ra10417d (PMC9060918; doi:10.1039/c8ra10417d)
Supplement: RA-009-C8RA10417D-s001 [file RA-009-C8RA10417D-s001.pdf]

Electronic Supplementary Material (ESI) for RSC Advances.
This journal is © The Royal Society of Chemistry 2019

Supporting information

Identifying the location of Cu ions in nanostructured SAPO-5 molecular sieves and its impact on the redox properties

Jörg Radnik,[*a] Thi Thuy Hanh Dang,[b] Suresh Gatla,[c,] Vikram Singh Raghuwanshi,[d,e†] Dragomir Tatchev, [f] and Armin Hoell [*e]

[a.] *Federal Institute for Material Research and Testing (BAM), Unter den Eichen 44-46, 12203 Berlin ,Germany.*

[b.] *National Key Laboratory of Petrochemistry and Refinery Technologies, Vietnam Institute of Industrial Chemistry, 2 Pham Ngu Lao, Hanoi, Vietnam.*

[c.] *ESRF-The European Synchrotron, 71, Avenue des Martyrs, 38000 Grenoble, France.*

[d.] *Humboldt University of Berlin, Institute of Chemistry, Brook-Tayler-Str. 2, 12489 Berlin, Germany*

[e.] *Helmholtz-Zentrum Berlin für Materialien und Energie, Hahn-Meitner Platz 1, 114109 Berlin, Germany*

[f.] *I Institute of Physical Chemistry – Bulgarian Academy of Sciences, Akad. G. Bonchev Str. Bl. 11, 1113 Sofia, Bulgaria.*

*Address correspondence to:

Joerg.Radnik@catalysis.de, hoell@helmholtz-berlin.de

Content:

- **X-ray diffraction (XRD)**
- **X-ray Absorption Spectroscopy (XAS)**
- **Anomalous Small-Angle X-ray Scattering (ASAXS)**
- **X-ray Photoelectron Spectroscopy (XPS)**

X-ray diffraction (XRD).

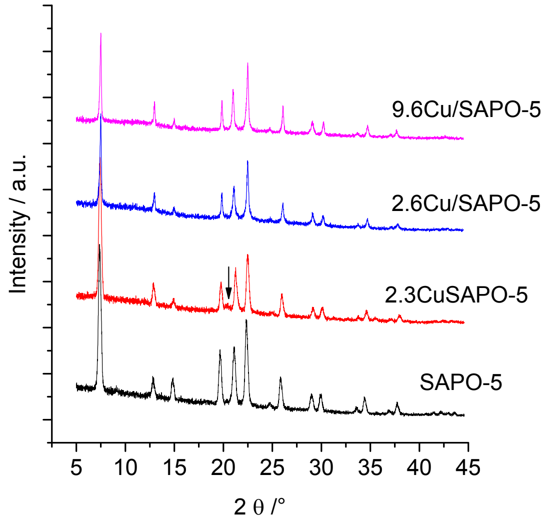

Fig.S1. XRD patterns of the three Cu containing samples and the unloaded SAPO-5. The arrow marked a weak reflection which cannot be correlated to SAPO-5. We propose triclinic $AlPO_4$ [PDF-No. 50-54] or SiO_2 tridymite [PDF-No. 83-1339].

X-ray absorption spectroscopy (XAS).

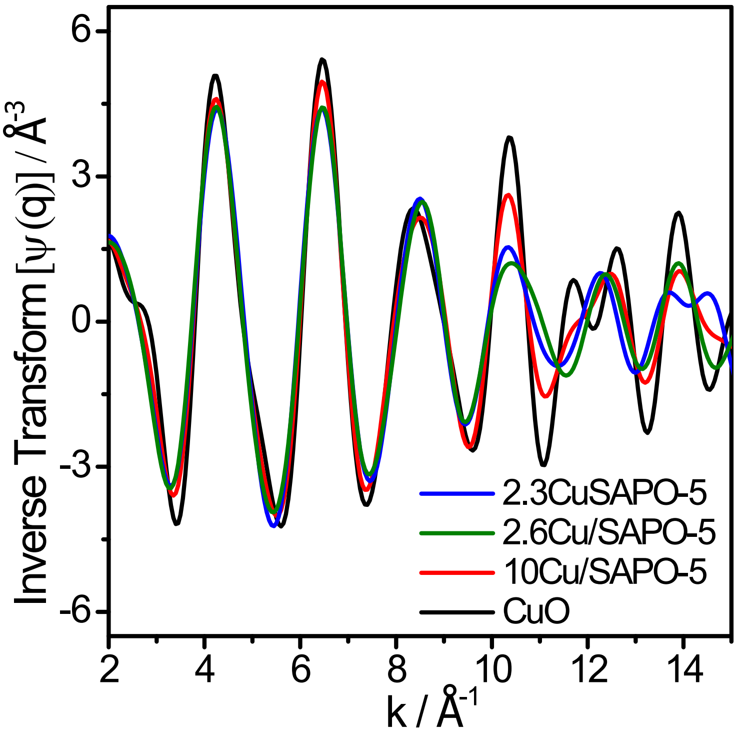

Fig. S2. k^3 weighted inverse Fourier transform of EXAFS spectra of 2.3CuSAPO-5, 2.6Cu/SAPO-5, 9.6Cu/SAPO-5 and CuO reference compound

Fig. S2 shows magnitude of the Fourier transform of the experimental EXAFS spectra of the samples and CuO. Fourier transform has been performed between k_{min} = 2 Å^{-1} to K_{max} = 15 Å^{-1} using a Kaiser-Bessel window. The peaks in the Fourier transform (FT) represent the contribution of the backscattering of photoelectron emitted at the Cu site from neighboring atoms providing the distribution of the atomic sites. The peaks appear in the FT, represent the raw data, and are slightly lower than the real atomic distances. This is due to back scattering effect, which is mainly depends on the atomic number of the backscatter. Therefore, shift correction need to be performed and the real values are shown in Table S1.

Table S1, Structural parameters fitted from EXAFS oscillations for CuO reference and the three Cu containing SAPO-5 samples.

**Sample**	**Atom pair**	**Coordination Number CN**	**Distance R (Å)**	**Debye-Weller-factor σ^2(Å^{-2})**	**Chemical shift ΔE (eV)**
**CuO**	Cu-$O_{xy,1}$	2	1.93 ± 0.02	0.008 ± 0.003	6
	Cu-$O_{xy,2}$	2	1.96 ± 0.01	0.003 ± 0.001	
	Cu-O_a	2	2.40 ± 0.20	0.060 ± 0.050	
	Cu-Cu_1	4	2.91 ± 0.01	0.007 ± 0.001	
	Cu-Cu_2	4	3.09 ± 0.01	0.007 ± 0.001	
	Cu-Cu_3	2	2.77 ± 0.02	0.013 ± 0.004	
**10Cu/SAPO-5**	Cu-$O_{xy,1}$	2	1.91 ± 0.03	0.008 ± 0.004	3
	Cu-$O_{xy,2}$	2	1.95 ± 0.02	0.003 ± 0.001	
	Cu-Cu_1	0.8	2.88 ± 0.01	0.005 ± 0.001	
	Cu-Cu_2	1.6	3.049 ± 0.003	0.010 ± 0.003	
**2.6Cu/SAPO-5**	Cu-$O_{xy,1}$	2	1.88 ± 0.03	0.006 ± 0.004	2
	Cu-$O_{xy,2}$	2	1.96 ± 0.03	0.003 ± 0.002	
**2.3CuSAPO-5**	Cu-$O_{xy,1}$	2	1.85 ± 0.01	0.004 ± 0.004	0.2
	Cu-$O_{xy,2}$	2	1.96 ± 0.01	0.002 ± 0.001	
	Cu-X(X= Si,Al orP)	2	2.90 ± 0.10	0.020 ± 0.020	
	Cu-X(X= Si,Al orP)	1.4	3.13 ± 0.04	0.010 ± 0.005	

Anomalous Small-Angle X-ray Scattering (ASAXS).

Small angle x-ray scattering (SAXS) is a method widely used in the analysis of nanoscale structures.4 In extension to SAXS, anomalous SAXS (ASAXS) allows element specific contrast variation between different phases in the sample and hence the possibility to determine chemical compositions as a function of particle dimension. The contrast variation in ASAXS is due to the dependency of the atomic scattering factor f (E) on the x-ray energy E, in particular, in the vicinity of X-ray absorption edges. Hence, for ASAXS experiments synchrotron x-ray sources have to be used where the x-ray energy can be continuously varied. The ASAXS curves have been measured at the 7T-MPW-SAXS beamline at the BESSY II synchrotron (HZB Berlin, Germany).

For the case of our systems we have to do Cu ASAXS

edge at E =8979 eV. The atomic scattering factor f (Z;E) can be written as:

f (E) = f0+ f ′(E)+i f ′′(E) (S1)

,where f0+ f ′(E) is the real part and f ′′(E) the imaginary part of the scattering amplitude. f0(Z)

depends only on the number of electrons in an atom, whereas f ′(E) and f ′′(E) depends on the

used X-ray energy. Thus the total amplitude f (E) deviates from the atomic number Z by varying the energy around an elemental absorption edge. The dispersion corrections f ′(E) and f ′′(E) are related to each other by the well known Kramers-Kronig relations and the values of the scattering factors were calculated by the procedure described by Cromer&Libermann.11

The four scattering contributions mentioned in the main paper were modeled as follows. Assuming independent scattering contributions the total scattering is

$$I(q,E) = I_K(q,E) + I_{mf}(q,E) + I_{pp}(q,E) + I_p(q,E) + I_{sp}(q,E) \qquad \text{(S2)}$$

where

$$I_K(q,E) = \frac{K(E)}{q^n} + B(E) \qquad \text{(S3)}$$

Is the contribution of the power law type at smallest angles, with K being a q-independent variable proportional to the scattering contrast, $\Delta\rho^2$, but B represents the background scattering;

$$I_{mf}(q,E)) = \int_0^\infty \Delta\rho_{mf}(E)^2 N(R) S(q,R) dR \qquad \text{(S4)}$$

with $N(R)$ being the log-normal size distribution of aggregate size and S(q,R,R_g) – the structure factor for mass fractal with Gaussian cut-off, R_g is the radius of gyration of the aggregate which for spherical shape is connected with the radius as $R = \sqrt{5/3}R_g$; $I_{pp}(q,E)$ and $I_p(q,E)$ fit the two peaks (pp – prepeak, p - peak) with Voigt functions

$$I_x(q,E) = A(E)\frac{\int_{-\infty}^{\infty}\frac{\exp(-u^2)}{\gamma^2/2\sigma^2 + ((q-q_c)/\sqrt{2}\sigma - u)^2}du}{\int_{-\infty}^{\infty}\frac{\exp(-u^2)}{\gamma^2/2\sigma^2 + u^2}du}\quad ; \qquad (S5)$$

and

$$I_{sp}(q,E) = \Delta\rho_{sp}(E)^2 N\, V(R)^2 \Phi(q,R)^2 \qquad (S4)$$

Represent scattering of a system of N spherical monodisperse particles which size was kept below 1.13 nm so that these particles could be able to reside in the channel of the SAPO-5 crystal structure. Here $\Phi(q,R)$ is the scattering form factor of a sphere. The x-ray energy dependence of the above equations is denoted by E. For each sample four scattering curves were measured at four x-ray energies, 8579, 8933, 8957 and 8975 eV. The data for 8579 eV (about 380 data points) were fitted with the equation (S2) containing up to 21 parameters. The so determined structure parameters were used to fit all four scattering curves by fixing the energy independent parameters and determining only the energy dependent ones. The data set per sample consisted of over 1500 points that were fitted with up to 24 free parameters. Relevant fit parameters are summarized in Table S2.

Table S2. Results obtained from the fitting of the ASAXS curves.

Sample		0 wt.% Cu	2.3 wt.% Cu		2.6 wt.% Cu	9.6 wt.% Cu
Method		hydrothermal	hydrothermal		impregnation	Impregnation
(100) peak		yes	yes		yes	yes
Prepeak		yes	no		Yes	no
ASAXS effect		no	no		no	Yes
Mass fractal	Dimension	2.79	2.77	2.78	2.89	2.86
	Mean R_g, nm	4.9	8.4	8.8	7.0	15.0
	Radius*, nm	7.8	13.3	13.9	11.0	23.9
	Dispersion	0.265	0.397	0.414	0.360	0.554
Large entities	Porod exponent	3.18	3.57	3.68	2.96	2.98
	Fractal dimension	2.82	2.43	2.32	2.96	2.98
Prepeak position, nm^{-1}		3.967	-	-	3.979	-
(100) peak position, nm^{-1} **		5.295	5.296	5.296	5.288	5.300
(100) peak position II*** (nm^{-1})		5.279	5.280	5.280	5.275	5.286
Spheres radius, nm		-	-	0.26	-	-
Reduced χ^2		0.953	1.273	1.253	0.813	0.547

*Assuming spherical aggregate
**Database position is 5.275 nm^{-1}
*** The peak tip only fitted with Gaussian function

Figure S3 left shows $K(E)/K_{max}$ where K_{max} is the maximum value of $K(E)$ for the used x-ray energies and Figure S3 right shows the same, but for $|\Delta\rho_{mf}(E)|^2$ of the aggregates. The two graphs contain also the data for the sample without Cu. Energy variations of the data of this sample are due entirely to systematic error and therefore represent a measure for the accuracy of the determination of the scattering contrasts. Errors obtained by the fitting procedure are too small to be relevant.

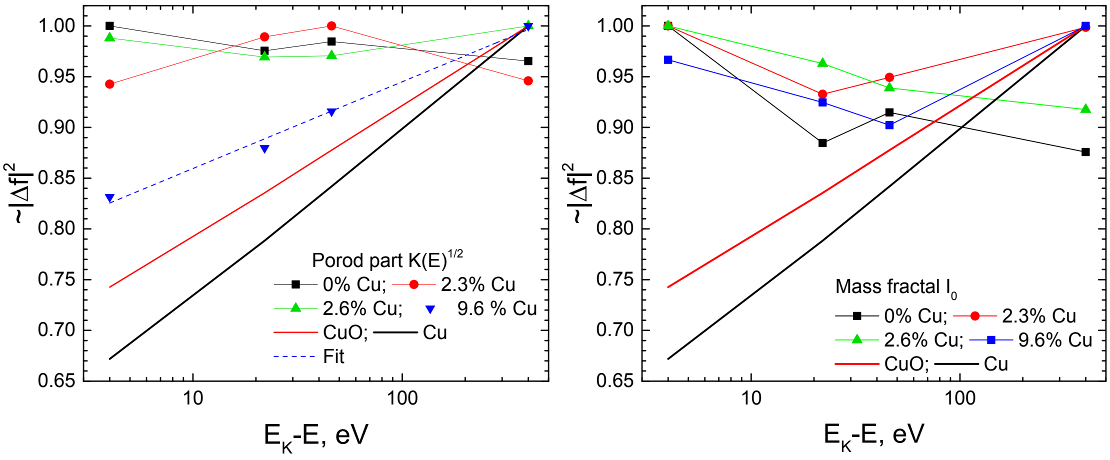

Figure S3. Energy dependence of variables proportional to the scattering contrast as determined from the power-law scattering part (left) and from mass fractal aggregates (right).

The high angle approximation for a core-shell particle is

$$K(E) = \left| [\Delta\rho_c - \Delta\rho_s(E)]\frac{R_c}{R_s} + \Delta\rho_s(E) \right|^2 \frac{2\pi S}{q^n} \tag{6}$$

where $\Delta\rho_{c,s}$ and $R_{c,s}$ are the scattering contrasts and the outer radii of the core and shell correspondingly. Under the condition $\Delta\rho_c \ll \Delta\rho_s$ it transforms to

$$K(E) = \left| \left[1 - \frac{R_c}{R_s} \right] \Delta\rho_s(E) \right|^2 \frac{2\pi S}{q^n} \tag{7}$$

and depends only on the shell contrast. In this case only we may expect the energy dependence of the Porod constant to follow the energy dependence of the shell contrast. In all other cases, the energy dependence will be influenced by the core contrast. As far as it is not possible to have 27 at% Cu in the core we conclude that Cu-enriched phase forms shell around large scattering entities. Most probably, that is CuO as detected by EXAFS but its contrast energy dependence is weaker (Figure S3 left) due to the core contrast influence.

X-ray photoelectron spectroscopy (XPS).

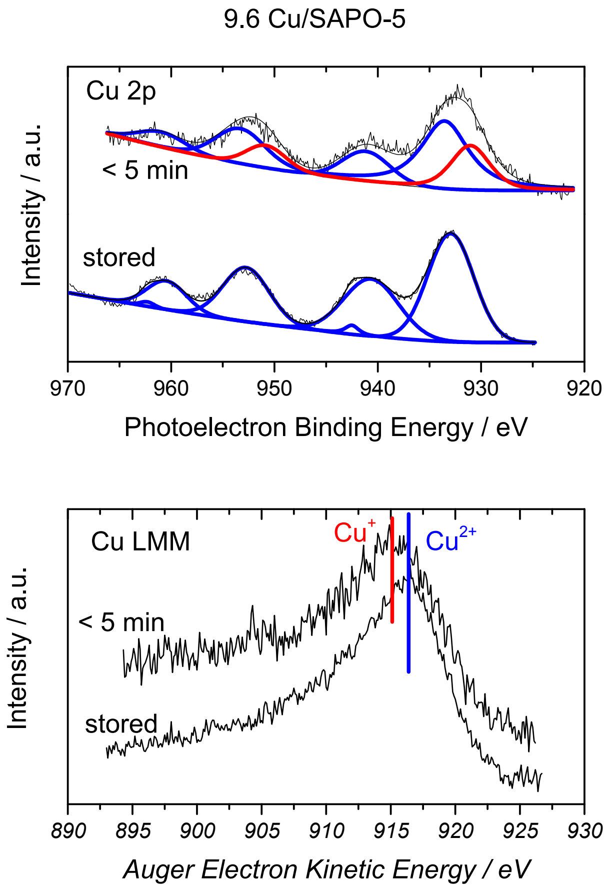

Fig.S4. Effect of storage period on the Cu states of the 9.6 Cu/SAPO-5. (stored = a few days)
